# Protective Effect of Biobran/MGN-3 against Sporadic Alzheimer's Disease Mouse Model: Possible Role of Oxidative Stress and Apoptotic Pathways

**DOI:** 10.1155/2021/8845064

**Published:** 2021-01-26

**Authors:** Mamdooh H. Ghoneum, Nesrine S. El Sayed

**Affiliations:** ^1^Department of Surgery, Charles Drew University of Medicine and Science, Los Angeles, California, USA; ^2^Department of Pharmacology and Toxicology, Faculty of Pharmacy, Cairo University, Cairo, Egypt

## Abstract

Alzheimer's disease (AD) is a debilitating and irreversible brain disease that affects an increasing number of aged individuals, mandating the development of protective nutraceuticals. Biobran/MGN-3, an arabinoxylan from rice bran, has potent antioxidant, antiaging, and immunomodulatory effects. The aim of the present study was to investigate the protective effect of Biobran against sporadic Alzheimer's disease (SAD). SAD was induced in mice via intracerebroventricular injection of streptozotocin (STZ) (3 mg/kg). STZ-treated mice were administered with Biobran for 21 days. The effects of Biobran on memory and learning were measured via the Morris water maze, novel object recognition, and Y-maze tests. Biomarkers for apoptosis, oxidative stress, and amyloidogenesis were measured using ELISA and western blot analysis. Histopathological examination was performed to confirm neuronal damage and amyloid-beta deposition. Biobran reversed the spatial memory deficit in SAD-induced mice, and it increased the expression of glutathione, reduced malondialdehyde, decreased IL-6, decreased intercellular adhesion molecule-1 (ICAM-1), and significantly increased nuclear factor erythroid 2-related factor 2 (Nrf2) and antioxidant response element (ARE). Moreover, Biobran exerted a protective effect against amyloid-beta-induced apoptosis via the suppression of both cleaved caspase-3 and the proapoptotic protein Bax and via the upregulation of the antiapoptotic protein Bcl-2. Furthermore, it reduced the expression of forkhead box class O proteins. It could be concluded from this study that Biobran may be a useful nutritional antioxidant agent for protection against SAD through its activation of the gene expression of Nrf2/ARE, which in turn modulates the apoptotic and amyloidogenic pathways.

## 1. Introduction

Alzheimer's disease (AD) is the most common neurodegenerative disorder characterized by progressive loss of memory and cognition. The appearance of oxidative stress markers is one of the hallmarks of AD; it leads to the build-up of amyloid deposits and neurofibrillary tangles and to the progression of the disease [1]. Oxidative stress is involved in many disorders, including Parkinson's disease, chronic inflammation, and AD [2]. Neurons produce energy at a high rate and show high oxygen consumption; they are at extremely high risk to oxidative damage from reactive oxygen species (ROS) [3]. Currently, the process by which amyloid-beta (A*β*) accumulation occurs in the central nervous system is uncertain, but the generation of ROS during A*β* self-aggregation is a potential mechanism by which A*β* may cause neuronal damage and death. This effect ultimately leads to synaptic membrane depolarization, excessive calcium influx, and mitochondrial impairment [4, 5]. One of the common regulators of the oxidative stress pathway in AD is the expression of the nuclear factor erythroid 2-related factor 2 (Nrf2). It is present mainly in the cytoplasm of the hippocampal neurons [6], and in AD animal models, the pathology of A*β* is linked with altered expression of Nrf2 target genes [7]. Nrf2 can act as a molecular switch in neurons that mediates the antioxidant system [8]. Active Nrf2 protects cells against oxidative injury by binding to the antioxidant response element (ARE) under oxidative stimuli and promoting antioxidative genes [9]. Therefore, the restoration of Nrf2 expression could alleviate cognitive impairment by protecting neurons against oxidative injury and decreasing A*β* accumulation [10].

Neuroinflammation plays an important role in AD pathogenesis through the production of inflammatory cytokines like IL-6, which is highly prevalent in AD [11, 12], and the intercellular adhesion molecule-1 (ICAM-1), which is highly expressed in the neuritic plaques in AD brains. ICAM-1 has been implicated in neurodegeneration through its role as an important mediator of immune cell activation and inflammatory response in AD [13]. ICAM-1 plays a key role in cell survival in the brain and induced the upregulation of proapoptotic proteins, Bax and cleaved caspase-3, and downregulation of the antiapoptotic proteins, Bcl-2 [14, 15]. This pathway has many downstream targets like the transcriptional factor forkhead box proteins of class O3a (FOXO3a), a factor that, when translocated to the nucleus, can trigger cell apoptosis. Evidence is growing that apoptotic markers can directly target FOXO3a and lead to cell apoptosis [16]. The family of FOXO proteins is extensively involved in the cell signal transduction of apoptosis and in oxidative stress. This effect is important in the survival of cerebral endothelial vascular cells [17], oxidative stress injury in mouse cerebellar granule neurons [18], and hippocampal neuronal injury [19]; and it can also lead to caspase 3-induced apoptotic death [20, 21].

STZ injection in the brain is linked to brain insulin resistance, neuroinflammation, oxidative stress, and deposition of A*β*, as well as tau protein aggregation leading to impairment in memory and learning functions that mimic sporadic Alzheimer's disease (SAD) in humans [22]. ICV-STZ injection induces the activation of microglial cells, which produce massive amounts of inflammatory mediators and free radicals that provoke neuronal damage [23].

Currently, there are no dietary supplements or prescribed medications for decreasing the risk of AD [24], and current FDA-approved treatments for AD are only symptomatic [25]. Biobran/MGN-3 is a denatured hemicellulose from rice bran that has shown promising effect as a natural adjuvant to existing immunotherapies for cancer [26, 27] through its antioxidant properties [28]. Biobran was shown previously to enhance natural killer cell activity in aged mice [29] as well as healthy elderly human subjects [30], with improvement in their health-related quality of life [31]. Few studies have been done on the beneficial effects of Biobran on aging and neurodegenerative diseases. So, the present study is aimed at investigating the possible protective effect of Biobran in a SAD model through the modulation of oxidative stress, amyloidogenesis, inflammation, and apoptotic pathways. Behavioral and biochemical experiments were performed to illuminate the mechanisms underlying its potential neuroprotective effect in the STZ model of SAD.

## 2. Material and Methods

### 2.1. Animals

Adult male Swiss albino mice, weighing 25-30 g, were used in the present study. Mice were obtained from the animal facility of the National Research Center, Cairo, Egypt, and they were housed 6 mice per cage. Mice were allowed to acclimate to their environment for a period of one week prior to the study. Animals were maintained in a controlled environment with constant temperatures (25 ± 2°C), light/dark cycles (12/12 h), and relative humidity (60 ± 10%). Animals were provided with a standard chow diet and allowed water ad libitum. This study complied with the Guide for the Care and Use of Laboratory Animals published by the US National Institutes of Health [32] and was approved by the Institutional Animal Care and Use Committee, Cairo University (CU-IACUC), approval number: CU-III-F-26-20. Animal discomfort and suffering was minimized as much as possible.

### 2.2. Drug Treatments

**Figure 1:**
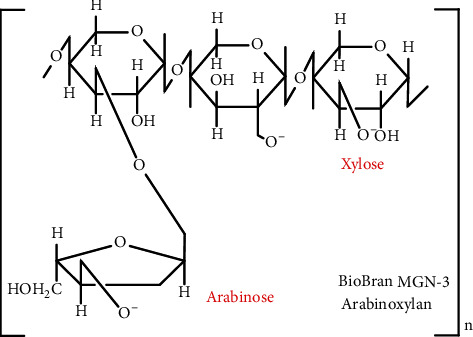
Chemical structure of Biobran/MGN-3.

STZ was purchased from Sigma-Aldrich Co. (St Louis, MO, USA) and dissolved in saline solution (0.9% NaCl). Biobran is a denatured hemicellulose that is extracted from rice bran by reacting rice bran hemicellulose with carbohydrate-hydrolyzing enzymes obtained from Shiitake mushrooms. Biobran's main chemical structure is arabinoxylan with an arabinose polymer in its side chain and a xylose in its main chain [26]. Daiwa Pharmaceutical Co. Ltd. (Tokyo, Japan) kindly provided Biobran. Biobran was prepared in saline (0.9% *w*/*v*) and was freshly prepared each day (Figure 1).

### 2.3. Acute Toxicity Study

Earlier studies have shown Biobran to be a nontoxic and safe agent. The Ames mutagenicity test is negative, Biobran's 50% lethal dose (LD50) is greater than 36 g/kg, and several toxicity studies have all confirmed the safety of Biobran in both humans and animals [27, 33]. 8-month-long periods of treatment of animals with Biobran as well as 5-year-long treatment for humans [34] have not resulted in any adverse side effects. In the present study, Biobran's acute toxicity in mice was assessed via the up and down procedure according to Guideline No. 423 from the Organization for Economic Cooperation and Development [35]. A starting dose of 2 g/kg Biobran was given to mice. For 24 hours, the mice were observed continuously for toxic symptoms, following which they were observed daily over an additional 20 days of maintenance.

### 2.4. Induction of SAD

SAD was induced in mice by ICV injection of STZ (3 mg/kg) into their lateral ventricle using the freehand procedure [36] updated by Warnock [37] for the avoidance of cerebral vein penetration. Thiopental (50 mg/kg, i.p.) was used to anesthetize the mice. The mouse head was secured using downward pressure above the ears, followed by insertion of the needle directly through the skin and skull into the lateral ventricle. Visualization of an equilateral triangle between the eyes and center of the skull was used to locate the bregma and target the lateral ventricle allowing the needle to be inserted at the following coordinates from bregma: 1 mm mediolateral, 0.1 mm anteroposterior, and 3 mm dorsoventral. The mice exhibited normal behavior approximately 1 minute after injection.

### 2.5. Experimental Design

**Figure 2:**
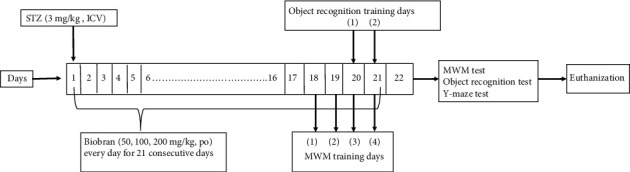
Experimental design.

Mice were randomly allocated into five groups, each containing 12 mice. Group 1, the sham control group, received one ICV saline injection followed by intraperitoneal (i.p.) saline injections for 21 consecutive days. Group 2 received STZ (3 mg/kg, ICV) once and served as the SAD model [38]. Group 3 received STZ (3 mg/kg, ICV), five hours later, it is followed by Biobran (50 mg/kg, i.p.) daily for 21 consecutive days. Group 4 received STZ (3 mg/kg, ICV), five hours later, it is followed by Biobran (100 mg/kg, i.p.) daily for 21 consecutive days. Group 5 received STZ (3 mg/kg, ICV), five hours later, it is followed by Biobran (200 mg/kg, i.p.) daily for 21 consecutive days. Once the treatment period ended, mice were given behavioral tests assessing cognitive functions (Figure 2).

### 2.6. Behavioral Assessments

#### 2.6.1. Object Recognition Test

To assess long-term memory and estimate cognition, we used the object recognition test. It is based on the concept of preference for novelty, which is the innate tendency of animals to exhibit an affinity for exploring a novel object rather than a familiar one [39]. This test was administered over three consecutive days. On day 1, each mouse was placed in a wooden box (30 × 30 × 30) dimension and was left for thirty minutes to adapt to the surroundings.

On day 2, two wooden cubes identical in shape, color, and size were placed at opposite corners in the box, 2 cm from the walls. Each mouse was placed in the box's middle and given ten minutes to explore the new objects. On day 3, one of the cubes was replaced by a novel object of different shape, size and color, and mice were each given five minutes to explore the box's objects. Objects and the arena were thoroughly cleaned with 70% ethanol between experiments with individual mice to ensure that their behavior was not guided by odor cues. Each mouse was faced toward the same wall at the beginning of each trial and was prevented from displacing the objects. Animals were video-recorded and measured for the following:

1. *Discrimination Index*. Time difference between the exploration of novel and familiar objects divided by the total exploration time
2. *Recognition Index*. Time spent exploring the novel object as a percentage of the total exploration time

#### 2.6.2. Morris Water Maze (MWM) Test

The MWM test was used to investigate spatial memory and learning [40]. The maze consisted of stainless-steel circular tanks (210 cm in diameter, 51 cm high) filled with water (25 ± 2°C) to a depth of 35 cm and divided into four quadrants. A black platform (10 cm wide, 28 cm high) was placed inside the target quadrant and submerged by 2 cm. The platform remained in the same location during training and testing procedures, and it was made invisible by coloring the water with a purple-colored nontoxic dye. Memory-acquisition trials (120 s/trail) were performed over four consecutive days, twice/day, with at least 15 min between trials. Animals were left free during each acquisition trial to locate the hidden platform. If the mouse located the platform, it was given 20 additional seconds for rest, while if the mouse did not reach the platform within 120 s, it was guided to the platform and allowed to rest there for 20 s. Mean escape latency (MEL) was calculated as the time taken by each mouse to find the hidden platform. After four acquisition trial days, mice were allowed 60 seconds to probe a pool in which the platform had been removed. Mice were put into the water in the Northeast position (Q4), and this is a fixed release point during the test. Measurements were made of the time each mouse spent in the target quadrant as an indicator of retrieval or memory.

#### 2.6.3. Y-Maze Test

The Y-maze is used to measure spatial working memory in rodents via the spontaneous alternation behavior (SAB) calculation [41]. Spontaneous alternation measures the ability of the animal to alternate its choice of arm entry on subsequent trials based on its memory of previous arm entries performed, which depends on the natural exploratory behavior of animals for new environments. The maze is a Y-shaped apparatus consisting of three arms, each one with the same dimensions, 35 cm long, 25 cm high, and 10 cm wide at 120° extending from a central platform. The apparatus was placed on the floor of the experimental room. The test was performed for 2 days. The first day was for the purpose of training; each mouse was positioned in the central platform and allowed to explore the maze freely for 8 minutes. The same procedure was followed on the second testing day, with the addition of manual recording of each arm entry, scored only when all four limbs of the mouse were inside the arm. After each session, the maze was cleaned with 70% ethanol to exclude any olfactory cues that might interfere with subsequent testing. An alternation was considered to have occurred if three successive different arms were entered during an overlapping triplet set. The percentage of spontaneous alternation activity (SAB%) was calculated as the “number of alternations consecutively” divided by “the total number of arm entries minus 2” and multiplied by 100.

### 2.7. Biochemical Assessments

Following the behavioral tests, animals (*n* = 12) were anesthetized using thiopental sodium (50 mg/kg, i.p.) and then euthanized by cervical dislocation. The brains were rapidly dissected on ice/salt mixture and washed with ice-cold saline. The hippocampi were homogenized in ice-cold saline to prepare 10% homogenates; these were split into several aliquots and stored at -80°C for estimation of the biochemical parameters (*n* = 6), western blot analysis (*n* = 3), and histopathological examination (*n* = 3).

### 2.8. Biochemical Measurements

#### 2.8.1. Determination of GSH and MDA

The hippocampal glutathione (GSH) content was measured spectrophotometrically using Ellman's reagent [42]. The peroxidation of hippocampal lipids was estimated by measuring malondialdehyde (MDA) levels via thiobarbituric acid reactive substances [43]. The results are expressed as mmol/mg protein.

#### 2.8.2. Determination of IL-6, ICAM-1, Cleaved Caspase-3, and Amyloid-*β*_1-42_

Hippocampal IL-6 and ICAM-1 levels were estimated using mouse ELISA kits purchased from RayBiotech Inc. (Norcross, Georgia, USA) and MyBioSource Inc. (San Diego, CA, USA), respectively. Cleaved caspase-3 and amyloid-*β*_1-42_ mouse ELISA kits were provided from Cusabio, Wuhan, China. The hippocampal levels of these markers were measured according to the manufacturer's instructions for each respective ELISA kit and expressed as their corresponding units to the tissue protein content determined by Salama et al. [44]. The ELISA assay measures the amount of sample by sandwiching it between two antibodies, one of which is precoated to the microtiter plate, and the other of which acts as a detector antibody. The microtiter plate provided in each kit was precoated with an antibody specific to each marker. Standards or samples are then added to the appropriate microtiter plate wells with a biotin-conjugated antibody preparation specific for the marker, and avidin conjugated to HRP is added to each microplate well and incubated. Then, a TMB substrate solution is added to each well. Only those wells that contain the protein, biotin-conjugated antibody, and enzyme-conjugated avidin will exhibit a change in color. The enzyme–substrate reaction is terminated by the addition of a sulfuric acid solution, and the color change is measured spectrophotometrically at a wavelength of 450 nm.

#### 2.8.3. Western Blot Analysis

Western blot is a method to quantify the expression level of specific proteins. Protein solutions were extracted from hippocampal tissues, *n* = 3. SDS-PAGE (10% acrylamide gel) was used to separate equal amounts of protein (averaging 20–30 *μ*g of total protein). Proteins were subsequently transferred to polyvinylidene difluoride membranes (Pierce, Rockford, IL, USA) with a Bio-Rad Trans-Blot system. Western blot immunodetection was performed by incubating the membranes at room temperature for 1 hour with blocking solution comprised of 20 mM Tris-Cl, pH 7.5, 150 mM NaCl, 0.1% Tween 20, and 3% bovine serum albumin. Membranes were incubated overnight at 4°C with one of the following primary antibodies: Nrf2 (catalog no: 31163), FOXO3a (catalog no: 1950), and *β*-actin (catalog no: 8227) obtained from Thermo Fisher Scientific Inc. (Rockford, IL, USA). Peroxidase-labelled secondary antibodies (1 : 1000; Novus Biologicals, Colorado, USA) were added after washing, followed by 1 h of membrane incubation at room temperature. Incubation with the substrate permits the detection of the amount of protein through optical documentation systems. The quantification of the band intensity can be used to determine specific protein levels in the tested tissues. Detection of a second “housekeeping” protein is necessary to control for variability in protein loading between samples. The band intensity was analyzed using the ChemiDoc™ imaging system with Image LabTM software version 5.1 (Bio-Rad Laboratories Inc., Hercules, CA, USA). The results are presented in arbitrary units after normalization to levels of the *β*-actin protein.

#### 2.8.4. Histopathological Analysis of Brain

*(1) H & E Staining*. The brain of 3 mice in each group was excised and fixed in 10% formol saline for 24 h. Washing was done in tap water then serial dilutions of alcohol (methyl, ethyl, and absolute ethyl) were used for dehydration. Specimens were cleared in xylene and embedded in paraffin at 56°C in a hot air oven for 24 h. Paraffin bees wax tissue blocks were prepared for sectioning at 4 *μ*m thickness by sledge microtome. The obtained tissue sections were collected on glass slides, deparaffinized, and stained by hematoxylin & eosin (H&E) stain for examination using a light electric microscope [45].

*(2) Congo Red Staining*. For Congo red staining, sections were stained with Congo red solution (0.2%) for 1 h and then counter-stained with hematoxylin solution. Plaques were observed and captured at 400 X magnification under a fluorescent microscope.

### 2.9. Statistical Analysis

Data are presented as mean ± S.D. Mean escape latency in Morris water maze trials was analyzed by repeated-measures analysis of variance (ANOVA). The remaining results were analyzed using one-way ANOVA followed by Tukey's multiple comparison test. Statistical analysis was performed using GraphPad Prism© software (version 6.01; Graph Pad Software, California, USA). For all the statistical tests, the level of significance was fixed at *P* < 0.05.

## 3. Results

### 3.1. Biobran's acute toxicity was investigated. We found no mortality, toxicity, or general behavior changes over a 24 h period at a dose of 2 g/kg

### 3.2. Neurobehavioral Analysis

The effects of STZ and Biobran (50, 100, and 200 mg/kg) on neurobehavioral tests were conducted within 24 h of the last day of Biobran injection.

#### 3.2.1. Mean Escape Latency (MEL)

**Figure 3:**
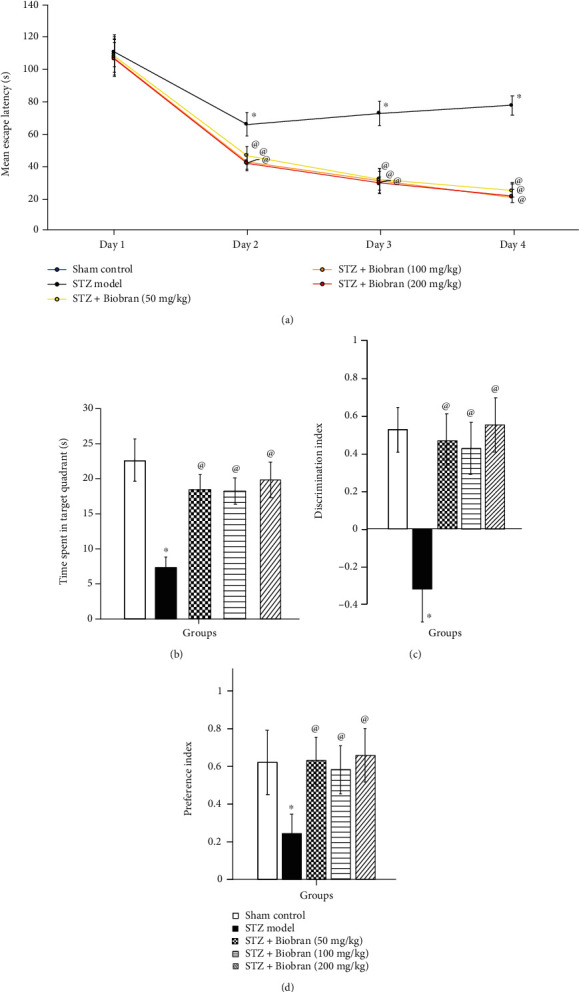
Effect of Biobran on the cognitive functions in MWM and NOR tasks in ICV-STZ injected mice. (a) Effect of Biobran on MEL in MWM. (b) Effect of Biobran on time spent in target quadrant in MWM. (c) Effect of Biobran on the discrimination index in NOR. (d) Effect of Biobran on the preference index in NOR. Values are expressed as mean ± SD; *n* = 12. Statistical analyses were performed using one-way analysis of variance (ANOVA) followed by the Tukey-Kramer post hoc test. ^∗^Significantly different from normal group at *p* < 0.05. ^@^Significantly different from ICV-STZ group at *p* < 0.05.

Figure 3(a) shows that the MEL in the MWM for STZ-treated mice was significantly higher (159%) than the MEL of the sham control mice at day 2, an effect that further increased at day 3 and 4. Mice treated with Biobran, on the other hand, had MEL values that were similar to the sham control starting on day 2.

#### 3.2.2. Time Spent in the Target Quadrant

Studies of Biobran's effect on time spent in the target quadrant of the MWM revealed that STZ-treated mice spent 32.6% of the time in the quadrant in comparison with the sham control, while animals treated with 50, 100, and 200 mg/kg of Biobran spent 81.7%, 81.0%, and 88.1% of the time, respectively, as compared to STZ-treated mice [*F* (4, 55) = 92.80, *P* < 0.0001] (Figure 3(b)).

#### 3.2.3. Discrimination and Preference Indices in the Novel Object Recognition (NOR) Test

The NOR test is used to examine the effect of STZ and Biobran on discrimination and preference indices. Administration of STZ in mice resulted in a decrease of the discrimination index compared to sham-control mice; on the other hand, it was increased significantly after Biobran administration in a dose-dependent manner. Additionally, the time ICV-STZ injected mice spent exploring the novel object was 39% of the time of the sham control group, which reflects a lower preference index. On the other hand, mice supplemented with Biobran were observed to prefer the novel object over the familiar object, normalizing the preference index in a dose-dependent manner (Figures 3(c) and 3(d)).

#### 3.2.4. Spontaneous Alternation Behavior in Y-Maze Task

The ICV-STZ group exhibited a significantly lower percentage of spontaneous alteration behavior, as compared to sham control mice. Treatment with different doses of Biobran resulted in a significant increase in the percentage of spontaneous alteration behavior, as compared to the ICV-STZ group [*F* (4, 55) = 37.90, *P* < 0.0001]. Thus, Biobran attenuated the STZ-induced impairment in short term memory as its administration caused a significant elevation in the percentage of spontaneous alteration behavior (Table 1).

### 3.3. Oxidative Stress Biomarkers

**Figure 4:**
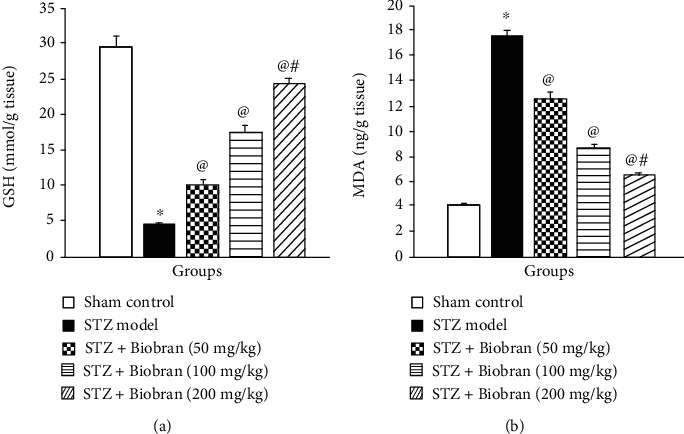
Effect of Biobran on MDA and GSH in ICV-STZ-injected mice. Values are expressed as mean ± SD; *n* = 6. Statistical analyses were performed using one-way analysis of variance (ANOVA) followed by the Tukey-Kramer post hoc test. ^∗^Significantly different from normal group at *p* < 0.05. ^@^Significantly different from ICV-STZ group at *p* < 0.05. ^#^Significantly different from Biobran (50 mg/kg) at *p* < 0.05.

The levels of MDA and GSH in the hippocampus were measured to investigate Biobran's protective effect on oxidative stress biomarkers. Administration of STZ resulted in a significant decrease of the GSH level by 15.5% compared to the sham control mice However, the administration of Biobran resulted in a significant increase in GSH content in a dose-dependent manner that was maximized at 82.8% at 200 mg/kg compared to ICV-STZ-injected mice [*F* (4, 40) = 771.4, *P* < 0.0001]. On the other hand, intracerebroventricular injection of STZ produced a significant increase in the MDA levels compared to sham control mice. Biobran supplementation for SAD mice reduced the MDA level in a dose-dependent manner compared to STZ-injected mice [*F* (4, 40) = 2180, *P* < 0.0001]. Moreover, Biobran (200 mg/kg) decreased significantly the MDA level as compared to Biobran (50 mg/kg) (Figure 4).

**Figure 5:**
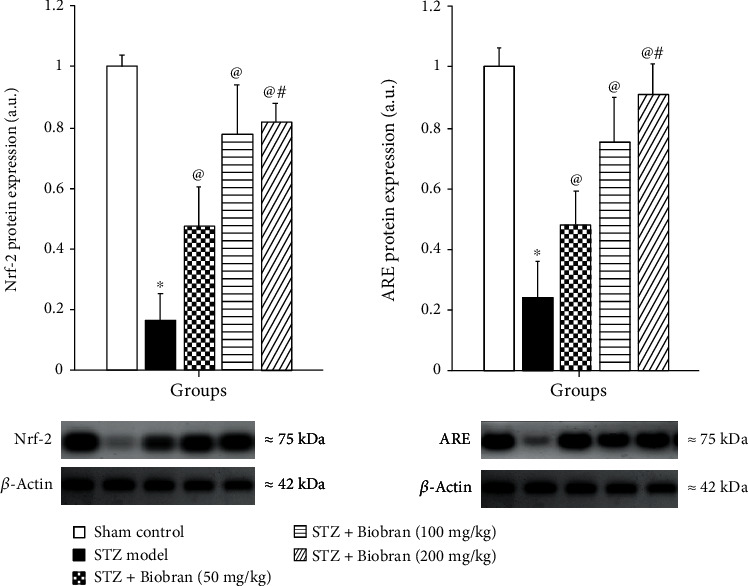
The effect of Biobran on Nrf2 and ARE in ICV-STZ-injected mice. Values are expressed as mean ± SD; *n* = 3. Statistical analyses were performed using one-way analysis of variance (ANOVA) followed by the Tukey-Kramer post hoc test. ^∗^Significantly different from normal group at *p* < 0.05. ^@^Significantly different from ICV-STZ group at *p* < 0.05. ^#^Significantly different from Biobran (50 mg/kg) at *p* < 0.05.

Oxidative stress was further studied via the expression of Nrf2 and ARE. Mice treated with STZ exhibited a significant decrease in hippocampal Nrf2 and ARE levels as compared to sham control mice, while exposure to Biobran resulted in a dose-dependent reversal of the expression of Nrf2 and ARE as compared to STZ-injected mice [*F* (4, 40) = 285.5, *P* < 0.0001], [*F* (4, 40) = 455.5, *P* < 0.0001], respectively, where a high dose of Biobran (200 mg) approximately returned Nrf2 and ARE levels to that of control (Figure 5).

### 3.4. Amyloid *β*_1-42_

**Figure 6:**
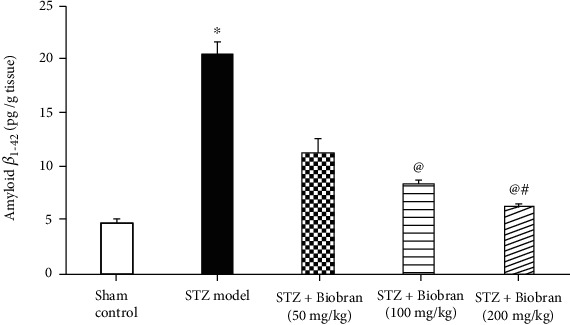
The effect of Biobran on A*β*_1-42_ in ICV-STZ-injected mice. Values are expressed as mean ± SD; *n* = 6. Statistical analyses were performed using one-way analysis of variance (ANOVA) followed by the Tukey-Kramer post hoc test, ^∗^Significantly different from normal group at *p* < 0.05. ^@^Significantly different from ICV-STZ group at *p* < 0.05. ^#^Significantly different from Biobran (50 mg/kg) at *p* < 0.05.

STZ-treated mice exhibited approximately a 4-fold increase in A*β* expression in comparison with the sham control. The A*β* levels were significantly decreased in STZ-treated mice after administration of Biobran to STZ injected mice [*F* (4, 40) = 2348, *P* < 0.0001]. The effect was dose-dependent, reaching the lowest level for 200 mg/kg (Figure 6).

### 3.5. Inflammatory Biomarkers

**Figure 7:**
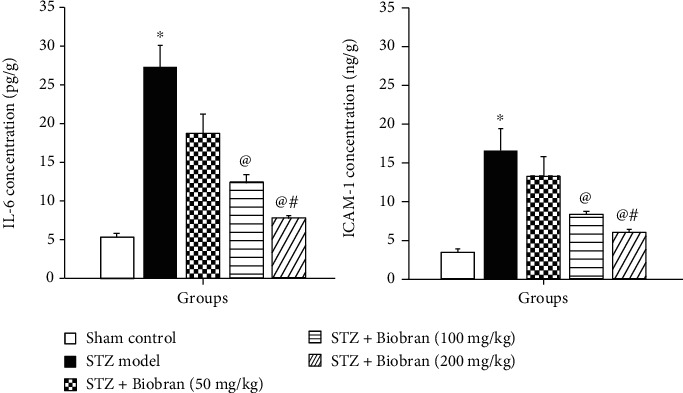
Effect of Biobran on IL-6 and ICAM-1 in ICV-STZ-injected mice. Values are expressed as mean ± SD; *n* = 6. Statistical analyses were performed using one-way analysis of variance (ANOVA) followed by the Tukey-Kramer post hoc test. ^∗^Significantly different from normal group at *p* < 0.05. ^@^Significantly different from ICV-STZ group at *p* < 0.05. ^#^Significantly different from Biobran (50 mg/kg) at *p* < 0.05.

In AD and many other diseases, autoimmune and inflammatory processes can be stimulated by IL-6. STZ-treated mice had a significant increase in IL-6 and ICAM-1 expression as compared to control mice. However, Biobran supplementation suppressed the levels of these markers in a dose-dependent manner that reached the control level at 200 mg/kg as compared to ICV-STZ mice [*F* (4, 40) = 646.2, *P* < 0.0001] and [*F* (4, 40) = 857.1, *P* < 0.0001], respectively (Figure 7).

### 3.6. Apoptotic Biomarkers

**Figure 8:**
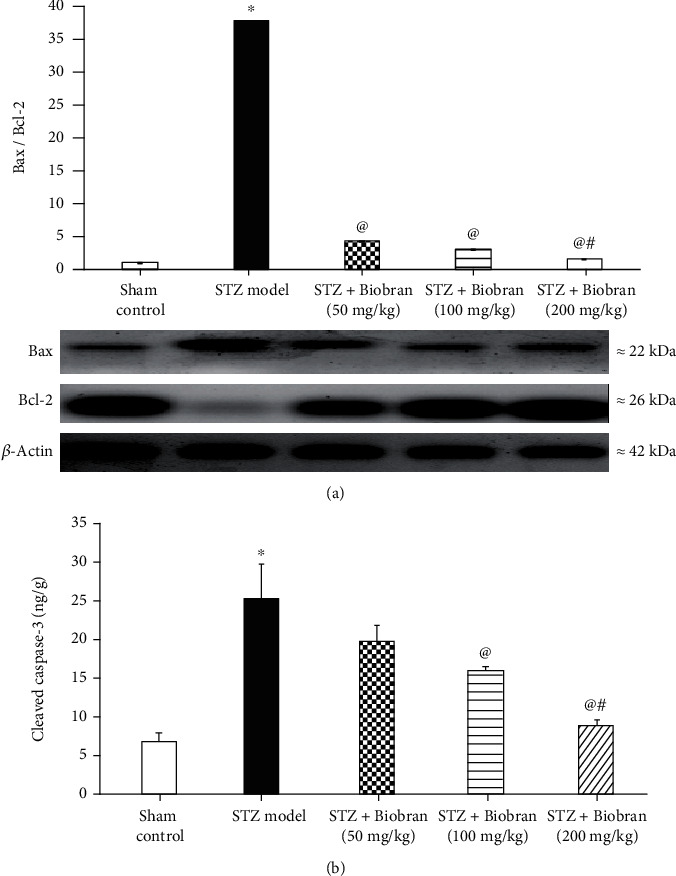
Effect of Biobran on the ratio of Bax to Bcl-2 (a) and cleaved Caspase-3 (b) in ICV-STZ-injected mice. Values are expressed as mean ± SD; *n* = 3. Statistical analyses were performed using one-way analysis of variance (ANOVA) followed by the Tukey-Kramer post hoc test. ^∗^Significantly different from normal group at *p* < 0.05. ^@^Significantly different from ICV-STZ group at *p* < 0.05. ^#^Significantly different from Biobran (50 mg/kg) at *p* < 0.05.

STZ-treated mice exerted a significantly increased expression of Bax while simultaneously showing a decrease in Bcl-2 expression in comparison to sham control. The Bax/Bcl-2 ratio in STZ-treated mice is 38 times greater compared to the ratio for sham control mice. In contrast, mice supplemented with Biobran demonstrated a dose-dependent reversal of Bax and Bcl-2 expression relative to STZ-treated mice. At the highest Biobran concentration, both Bax and Bcl-2 expression were comparable to the sham control level, with the Bax/Bcl-2 ratio being only 1.5 times as high as the sham control (Figure 8(a)). A similar effect was seen for cleaved caspase-3 expression. In STZ mice, cleaved caspase-3 levels increased approximately 5-fold relative to control. Following supplementation with Biobran, STZ-treated mice exhibited a gradual decrease in cleaved caspase-3 expression in a dose-dependent manner [*F* (4, 40) = 1728, *P* < 0.0001] (Figure 8(b)).

**Figure 9:**
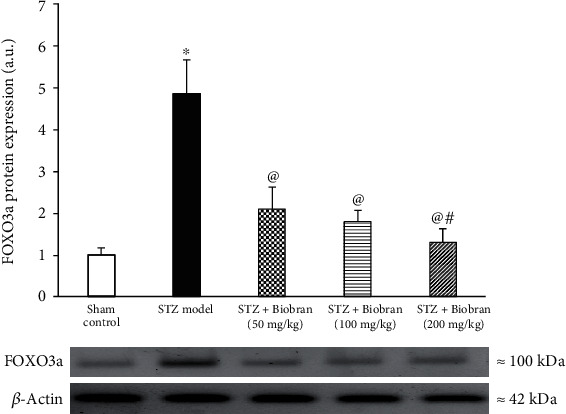
Effect of Biobran on FOXO3a in ICV-STZ injected mice. Values are expressed as mean ± SD; *n* = 3. Statistical analyses were performed using one-way analysis of variance (ANOVA) followed by the Tukey-Kramer post hoc test. ^∗^Significantly different from normal group at *p* < 0.05. ^@^Significantly different from ICV-STZ group at *p* < 0.05. ^#^Significantly different from Biobran (50 mg/kg) at *p* < 0.05.

We furthermore analyzed the expression of FOXO3a protein. A significant increase by 5-fold in the expression of FOXO3a was revealed in STZ-treated mice relative to control. Following supplementation with Biobran, FOXO3a expression in STZ-treated mice decreased in a dose-dependent manner, with the highest dose (200 mg) nearly bringing FOXO3a expression back to the level of control compared to STZ-injected mice [*F* (4, 25) = 670.3, *P* < 0.0001], respectively] (Figure 9).

### 3.7. Histopathology Analysis

Brains of sham control mice showed normal structure of the brain tissue including the cerebral cortex and the hippocampus. Microscopic examination of the STZ group showed several histopathological changes in the brain tissue. The cerebral cortex showed numerous scattered dark degenerated neurons that were associated with neuronophagia and diffuse gliosis. The hippocampus showed multifocal haemorrhagic areas with dark degenerated neurons in the CA3, CA4, and DG regions.

Mice treated with Biobran (50 mg/kg) ameliorated the effects of STZ. Sections of the cerebral cortex showed decreased numbers of dark neurons with healthy neurons in most examined sections. The hippocampus showed normal neurons in the various neurological regions.

Mice treated with Biobran (100 mg/kg and 200 mg/kg) showed normal histological structure of the cerebral cortex except for few degenerated neurons and neuronophagia. The hippocampus appeared apparently normal.

**Figure 10:**
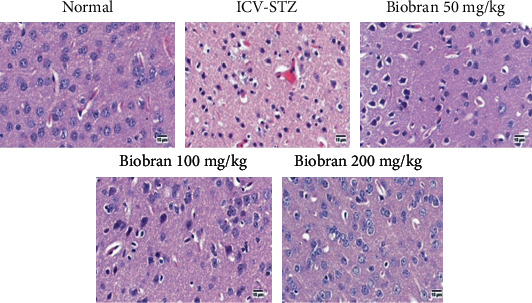
Effect of Biobran (50, 100, and 200 mg/kg) on histopathological changes in the cerebral cortex of ICV-STZ injected mice, *n* = 3. Control: showing normal histological structure of the cerebral cortex of normal mice; ICV-STZ: showing diffuse gliosis in the cerebral cortex admixed with numerous degenerated neurons in the injected mice; Biobran 50 mg/kg: showing moderate number of degenerated neurons; Biobran 100 mg/kg: showing few injured neurons; Biobran 200 mg/kg: showing apparently normal cerebral cortex structure.

**Figure 11:**
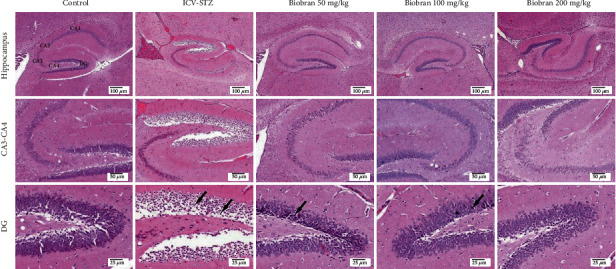
Effect of Biobran (50, 100, and 200 mg/kg) on histopathological changes in the hippocampus of ICV-STZ-injected mice, *n* = 3. Control: showing normal histological structure of different CA regions and DG of the hippocampus of normal mice; ICV-STZ: showing edema of CA1, CA2, and DG with dark-degenerated neurons in CA3, CA4, and DG (arrows) in the hippocampus and congestion in the surrounding brain parenchyma; Biobran 50 mg/Kg: showing dark degenerated neurons in the CA1, CA4, and DG regions (arrow) of the hippocampus; Biobran 100 mg/Kg: showing apparently normal neurons in the hippocampus with few scattered neurons in CA4 and DG (arrow); Biobran 200 mg/Kg: showing apparently normal neurons in the hippocampus.

The administration of Biobran showed no histopathological alterations in the brain tissue with normal structure of the cerebral cortex and the hippocampus (Figures 10 and 11).

**Figure 12:**
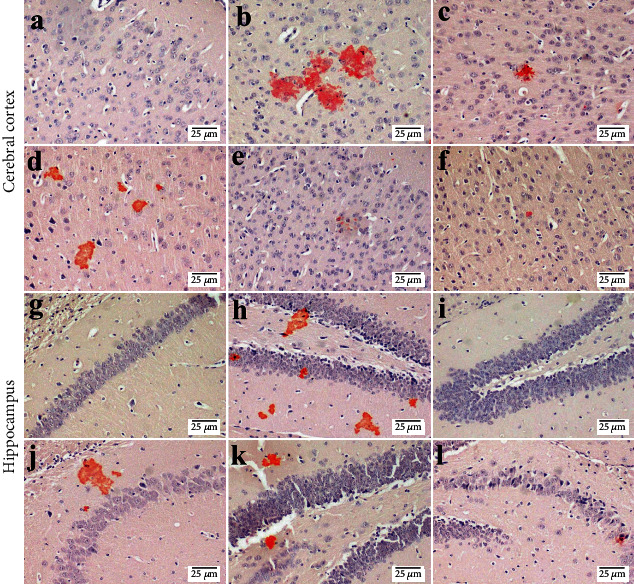
Congo red—brain-stained sections of mice for amyloid plaques visualization in the cerebral cortex and hippocampus (*n* = 3). (a, g, i) represent the normal group showing no deposition of amyloid plaques. (b, h) represent the STZ group showing multifocal deposition in the cerebral cortex in the cerebral cortex and the hippocampus, respectively. (c, d, j) represent Biobran (50 mg/kg) in STZ-injected mice, showing multifocal scattered plaques. (e, k) represent Biobran (100 mg/kg) in STZ-injected mice, showing few deposition in the cerebral cortex and multifocal deposition in the hippocampus. (f, l) represent Biobran (200 mg/kg) in STZ-injected mice showing minute deposition of amyloid plaques.

The number of amyloid plaques was investigated in different experimental groups through visualization with Congo red stain. Normal mice showed no amyloid deposition in the brain sections. Meanwhile, ICV injection of STZ showed multifocal deposition of the amyloid deposition in the brain tissue especially in the inflammatory lesion that showed focal gliosis. The administration of Biobran (50 mg/kg) resulted in a marked reduction of the number of amyloid plaques in the brain tissue. Moreover, mice that received Biobran (100 mg/kg) showed few amyloid plaques, and mice that received Biobran (200 mg/kg) showed an absence of amyloid plaques in most examined brain tissue (Figure 12).

## 4. Discussion

The current study evaluated the protective effect of Biobran/MGN-3 against STZ-induced SAD in mice. Biobran, a natural biological response modifier, has been shown to possess antiaging [29–31] and antioxidant [37] properties. Biobran is proved previously to exhibit potent immunomodulatory functions [26, 27, 46–48] and exert beneficial effects against cancer, viruses, and microbes [49–52].

In the present study, STZ-treated mice were unable to discriminate between novel and familiar objects, as demonstrated by the NOR task. The ICV-STZ group revealed marked deterioration in memory and learning functions as observed in the Morris water maze and manifested by a significant decrease in the time spent in the target quadrant as well as in the Y-maze tests demonstrated by a significant decline in the spontaneous alternation behavior. These findings are in agreement with previous studies reporting that ICV-STZ injection was implicated in decreased spontaneous alternation behavior in the Y-maze test and a decline in spatial learning and reference memory in Morris water maze trials as well as the test day [53, 54]. This indicates obvious memory and learning deficits in these mice. The ICV injection of STZ is a well-known model of sporadic Alzheimer's disease in rodents with similar progressive pathology of AD as in the human brain [55–57]. However, it was of great interest to note that Biobran supplementation prevented the STZ-induced impairments of short-term and spatial memory. In a dose-dependent manner, Biobran reduced the MEL time, extended the time spent in the target quadrant, and reversed the discrimination and preference indices as well as decreasing the spontaneous alternation behavior in the Y-maze task.

Coherent to the aforementioned findings, it was found that the cognitive dysfunction exerted a positive impact on the amyloidogenic and oxidative stress pathways. A*β* peptide is one of the hallmarks of AD causing neuronal loss in the brain and resulting into deficits in memory and learning. Previous studies revealed that antioxidant compounds could be promising therapeutic or preventive interventions for AD patients because they inhibit A*β* fibril formation and protect the brain from A*β* neurotoxicity [58]. In the current study, Biobran exerted a significant antioxidant effect in the model of SAD, which is in agreement with previous studies that revealed Biobran's antioxidant activity against murine solid Ehrlich carcinoma [28], as well as its ability to significantly alleviate the increase in MDA content and prevent the irradiation-induced depletion of GSH in mice spleens [59].

ROS generation caused by mitochondrial oxidative phosphorylation can have profound effects on cellular functions and result in the initiation of many diseases, including aging [60] and AD [2, 61]. During oxidative stress, ROS can lead to neuronal synaptic dysfunction [62, 63] and may cause neuronal damage and death during A*β* self-aggregation [64]. Biobran has been shown previously to upregulate the oxidative stress in the liver and to inhibit the levels of these biomarkers including MDA, total free radicals, and nitric oxide in murine Ehrlich carcinoma [28]. This suggests that Biobran induces oncostatic activity by providing protection against oxidative stress, modulating lipid peroxidation, and enhancing the antioxidant defense system. Moreover, it was reported previously that Nrf2 is suppressed in AD patients' neurons [65], which is in harmony with the results of the present study. For AD animals, there is a decrease in Nrf2 expression, as well as in the expression of the Nrf2/ARE pathway's target genes [66]. Altered expression in Nrf2 is associated with cognitive deficits and impaired spatial memory in mouse models of AD [6] and a deficiency in Nrf2 results in vulnerability to oxidative stress [67], phosphorylated-Tau [68], and enhanced autophagic dysfunction [7]. On the other hand, it was revealed previously that neurons can be protected against A*β* pathology and oxidative proteotoxic stress by the upregulation of the Nrf2/ARE pathway [69, 70]. Several studies have shown that neuropathological changes such as AD and Parkinson's are also associated with faulty inflammatory processes such as increased expression of the proinflammatory cytokine IL-6 in the brain [11, 12]. In the current study, IL-6 and ICAM-1 were significantly increased after STZ administration, but Biobran supplementation caused a significant decrease in the levels of these biomarkers. Interestingly, a recent clinical study revealed the increased concentrations of IL-6 and ICAM-1 in AD patients' cerebrospinal fluid (CSF) [71, 72]. These effects were more evident in patients with abnormal CSF A*β* levels, indicating that, in the presence of A*β* pathology, associations between cerebrovascular, neurodegenerative, and neuroinflammatory processes may be aggravated and contribute to tau aggregation, leading to cognitive impairment and disease progression [71]. Therefore, focusing on these biomarkers offers potential targets for novel therapeutic interventions. In the present work, the administration of Biobran significantly inhibited the levels of ICAM-1 and IL-6 in the hippocampi of SAD-induced mice with significant accumulation of amyloid plaques.

In the present study, Biobran exerted an antiapoptotic effect against STZ in a dose-dependent manner. This effect could be due to suppression of cleaved caspase-3 as well as the proapoptotic protein Bax and through the downregulation of the antiapoptotic protein Bcl-2. It has been suggested previously that A*β* activates the neuronal apoptotic pathway via its accumulation in the mitochondrial membrane and impairment of mitochondrial function [73]. The membrane of mitochondria becomes permeable during mitochondrial apoptosis, and ROS gets released [74]. Apoptogenic proteins such as cytochrome c can thereby be produced, and proapoptotic factors can be introduced into the cytosol from the mitochondria, ultimately activating procaspases and inducing apoptosis [75]. Neuronal loss can be caused by mitochondrial dysfunction via the regulation of proapoptotic proteins like caspase-3 and Bax and antiapoptotic proteins like Bcl-2 [14, 15, 20]. The ability of Biobran to exert a protective effect against STZ-induced apoptosis is in accordance with our earlier studies showing that Biobran treatment upregulated Bax expression, activated caspase-3, and downregulated Bcl-2 expression; these well-established molecular events in apoptosis have shown that Biobran can protect against glandular stomach carcinogenesis in rats [49], inhibit hepatocarcinogenesis in rats [50], and enhance fractionated X-ray irradiation's anticancer effects for Ehrlich solid tumor-bearing mice [59].

The effect of Biobran on FOXO protein expression in STZ-injected mice hippocampi was also examined. FOXO proteins have a range of biological functions. They are present throughout the body and are selectively expressed in the nervous system. The complex interaction between signal transduction pathways and FOXO proteins in the presence of oxidative stress can significantly impact apoptosis and autophagy [18, 20, 76]. Under oxidative stress conditions, autophagy can be induced by FOXO proteins along with the promotion of cell survival [64]. STZ-injected mice showed significantly higher levels of the FOXO protein expression. Biobran decreased significantly these values in a dose-dependent manner. This demonstrates Biobran's protective effect against FOXO-mediated apoptosis in STZ-treated mice. Recently, it was reported that Biobran/MGN-3 is a promising psychoneuroimmune modulatory agent that could improve the quality of life in healthy old adults [31].

Histopathological analysis using H&E and Congo red stainings further revealed the protective effect of Biobran against STZ-induced neuronal damage in the cerebral cortex and hippocampal sections of mice. Treatment with Biobran reduced the neuronal toxicity observed in STZ-injected mice, with fewer eosinophilic-stained neurons and more healthy neurons with prominent nuclei. This indicates that Biobran could act as a potential candidate to attenuate neurodegeneration and preserve cognitive functions. Hippocampal sections of Biobran-treated groups also exhibited a dose-dependent protective effect against A*β* plaque formation. These positive histological effects of Biobran are in agreement with our behavioral and biochemical assessments. The highest dose of Biobran exerted better protection compared to the low and moderate doses.

## 5. Conclusions

Biobran exerts a dose-dependent protective effect against sporadic AD. This effect is achieved through the targeting of the Nrf2/ARE antioxidant signaling that modulates amyloidogenesis as well as the Bcl2/Bax/caspase-3 pathway. To our knowledge, the present study is the first to investigate the protective effect of Biobran against SAD. Our findings suggest that Biobran's activity is able to reduce A*β* generation and promotes cognitive function recovery. They may suggest the possible applicability of Biobran in clinical trials of human subjects in the management of SAD.

## Figures and Tables

**Table 1:** Effect of Biobran (50, 100, and 200 mg/kg) on spontaneous alternation behavior in Y-maze task in ICV-STZ-injected mice. Values are expressed as mean ± SD; *n* = 12. Statistical analyses were performed using one-way analysis of variance (ANOVA) followed by the Tukey-Kramer post hoc test. ∗ Significantly different from the normal group at *P* < 0.05, ^@^Significantly different from the ICV-STZ group at *P* < 0.05.

Groups	Sham control	STZ model	STZ + Biobran (50 mg/kg)	STZ + Biobran (100 mg/kg)	STZ + Biobran (200 mg/kg)
% Spontaneous alternation	68.29 ± 2.07	^∗^35.72 ± 1.72	^@^55.79 ± 1.55	^@^59.32 ± 1.68	^@^65.87 ± 2.05

## Data Availability

The data of the present study including the figures and western blot analysis used to support the findings of this study are included within the article.
